# A brief review and case report of pheochromocytoma misdiagnosed as allergic vasculitis with bilateral lower extremity ulcers: a 24-year clinical course

**DOI:** 10.3389/fendo.2026.1773861

**Published:** 2026-04-10

**Authors:** Rui Li, Ziyi Cao, Heting Wei, Jie Han, Shuping Guo, Le Zhang, Xiaoqing Lang, Hongye Liu, Hongzhou Cui

**Affiliations:** 1 The First Clinical Medical College of Shanxi Medical University, Taiyuan, Shanxi, China; 2 Department of Dermatology, First Hospital of Shanxi Medical University, Taiyuan, Shanxi, China

**Keywords:** allergic vasculitis, atypical presentation, catecholamine, diagnostic delay, limb ulcers, pheochromocytoma

## Abstract

We report a 36-year-old male with pheochromocytoma presenting solely as progressive bilateral lower limb necrosis for 24 years, lacking classic symptoms (hypertension, headache, palpitations). Misdiagnosed as allergic vasculitis, he developed atrophic scars and toe necrosis despite immunosuppression. Elevated catecholamines and a 4.8 cm adrenal mass confirmed the diagnosis. Postoperatively, ulcers healed, but toe amputation was needed. This case highlights diagnostic pitfalls of atypical pheochromocytoma, emphasizing dynamic biomarker monitoring for early detection.

## 1 Introduction

Pheochromocytoma is a rare tumor originating from chromaffin cells of the adrenal medulla, typically presenting with paroxysmal hypertension, palpitations, sweating, and headache. However, cases with atypical manifestations (e.g., isolated peripheral vascular lesions) are easily misdiagnosed, leading to delayed treatment.

## 2 Case description

A 36-year-old man was admitted with a 24-year history of painful, recurrent ulcerations and necrosis involving both lower limbs. The initial episode occurred 24 years earlier, when erythema and pain developed in the left foot after exposure to alternating heat and cold. Over the following months, the lesions steadily extended to both legs and feet, culminating in repeated ulceration. Despite multiple courses of antimicrobial therapy, clinical response remained poor. Two years after onset, histopathology of a skin-biopsy specimen demonstrated leukocytoclastic (allergic) vasculitis. Systemic corticosteroids, antibiotics, and vasodilatory agents induced only transient remission; exacerbations recurred one to two times per year, predominantly after cold exposure and during spring and autumn. Each flare left residual hyperpigmentation and atrophic scarring. The patient was a long-term smoker but reported no relevant family history or other systemic manifestations.

**Figure 1:**
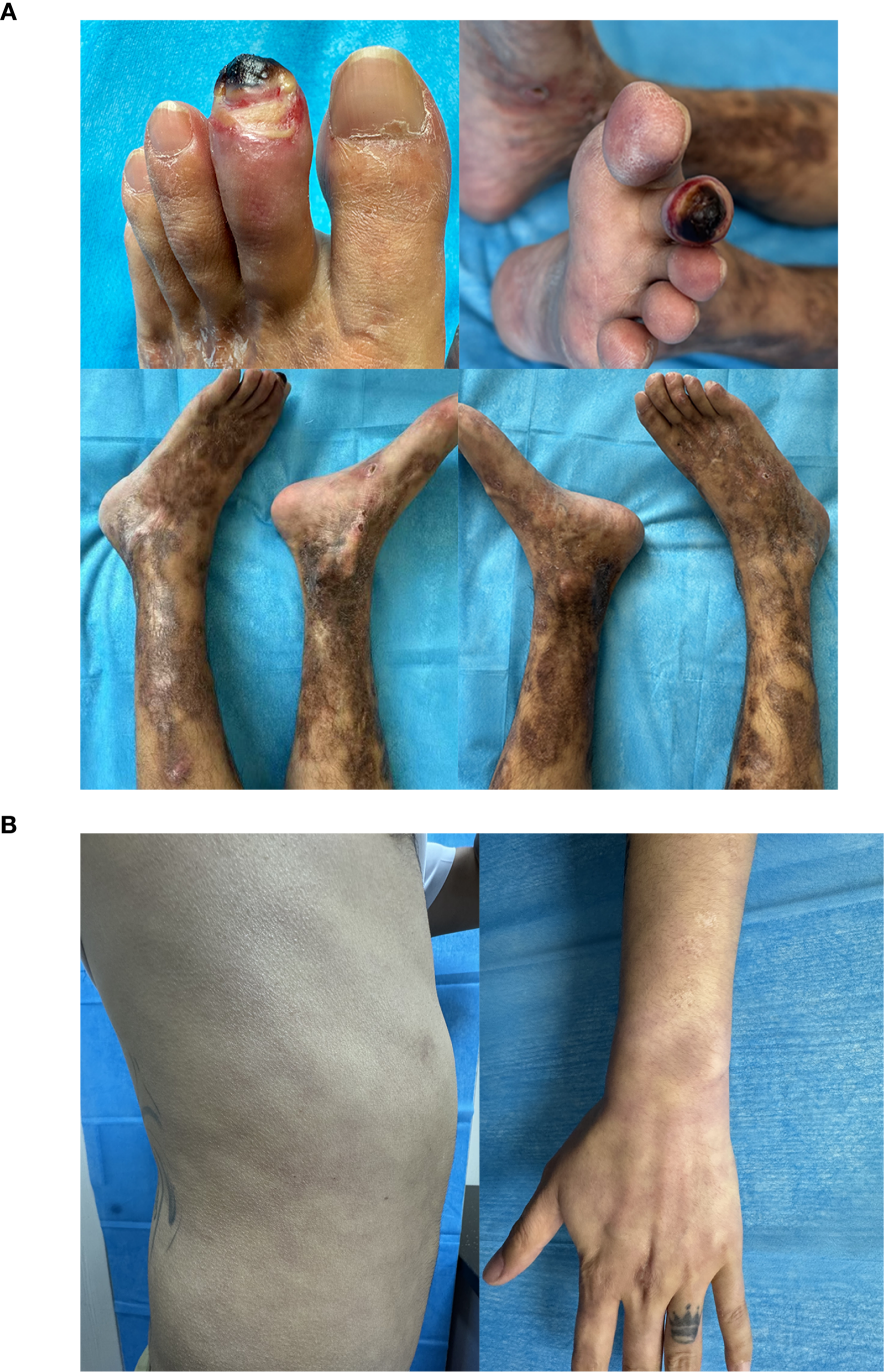
**(A)** Long-term immunosuppression and antibiotic therapy failed to prevent recurrence, resulting in atrophic scarring of both lower extremities and irreversible necrosis of the tip of the second toe of the left foot. **(B)**. Transient symmetrical cyanosis of the trunk and limbs occurs after cold exposure.

The dermatological examination is as shown in the figure (**Figures 1a, b**). Laboratory results showed that complete blood count, vasculitis screening, and autoimmune panels were all negative, with no evidence of coagulopathy. A repeat skin histopathological examination after admission revealed nonspecific vasculitis. Duplex ultrasonography of the arteries and veins in both lower limbs showed no significant abnormalities. CT angiography (CTA) of both lower limbs was unremarkable. Contrast-enhanced abdominal CT revealed a suspected pheochromocytoma, measuring approximately 4.8 × 3.7 cm. Plasma norepinephrine (NE) was elevated at 1.670 ng/mL (supine reference range: 0.112–0.685 ng/mL), and plasma dopamine (DA) was increased to 0.360 ng/mL (supine reference: <0.010 ng/mL). Serum normetanephrine (NME) was also markedly elevated at 0.710 ng/mL (reference: <0.050 ng/mL). Twenty-four-hour urinary norepinephrine excretion was 336.57 μg/day (reference range: 15.00–80.00 μg/day). To explore the potential association between symptom fluctuation and catecholamine levels, we performed a prospective dynamic monitoring of plasma free normetanephrine: fasting venous blood samples were collected during 3 episodes of typical acute pain (with aggravated ulcers and severe limb distension/pain) and 2 periods of symptom remission (with reduced pain and relatively stable wounds) after admission, and quantitative detection was conducted for each sample. Repeated detection verified that the peak plasma free normetanephrine level during acute pain episodes was 17 times higher than that during remission periods. Adrenocorticotropic hormone (ACTH), sex hormones, and parathyroid hormone levels were all within normal limits. During an out-of-office examination after admission, the patient accidentally discovered transient symmetrical cyanosis on the trunk and limbs after cold exposure. (**Figure 1b**) The patient denied any history of paroxysmal palpitations, excessive sweating, dizziness, or headaches—symptoms typically associated with pheochromocytoma.

## 3 Discussion and conclusion

**Figure 2:**
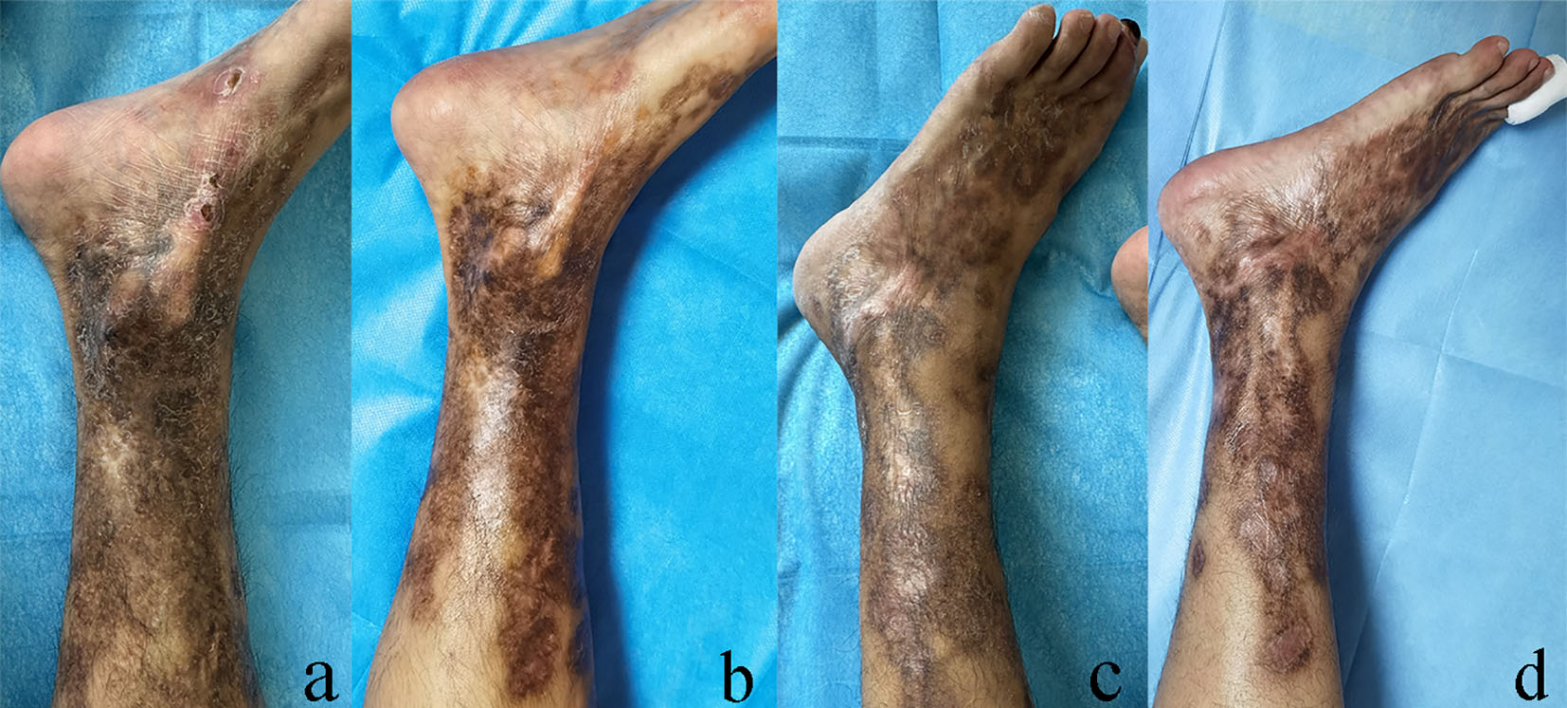
Most of the lower limbs are left with black-brown patches of varying sizes, reddish atrophic scars, and scattered old ulcers. **(a, c)** Ulcer healing 1 month after surgery, no new ulcer appeared; some dark spots faded and disappeared, especially the edges of the black spots and the edges of the soles of the feet. **(b, d)** However, due to irreversible microvascular damage, the toe needs to be amputated. **(d)** The left side of each group of pictures is the picture before treatment, and the right side is the picture after treatment.

The patient underwent laparoscopic right adrenalectomy, and histopathological analysis confirmed the diagnosis of a benign pheochromocytoma. At the follow-up one month after surgery, the patient’s bilateral lower extremity ulcers had completely healed, pain relief, significant improvement in peripheral circulation, partial hyperpigmentation had faded, and blood and urine catecholamines had returned to normal (**Figure 2**). However, due to irreversible microvascular damage, the patient underwent a distal amputation of the second toe of the left foot, and is currently receiving clinical follow-up.

To summarize the clinical characteristics and prognostic outcomes of atypical pheochromocytoma presenting with isolated peripheral vascular lesions, a systematic literature review was conducted following the PRISMA guidelines. Electronic databases including PubMed, Embase, Web of Science, and CNKI were searched from their inception to December 2025, with the key search terms: “pheochromocytoma” OR “phaeochromocytoma” AND “limb ulcer” AND “peripheral vascular lesion” AND “atypical presentation” AND “no hypertension”. The inclusion criteria were: (1) patients diagnosed with pheochromocytoma; (2) clinical manifestations dominated by isolated peripheral vascular lesions/limb ulcers without classic hypertensive symptoms (headache, palpitations, sweating); (3) complete clinical data including diagnosis, treatment and follow-up outcomes. The exclusion criteria were: (1) cases with incomplete clinical information; (2) patients combined with malignant tumors, primary vascular diseases or other systemic autoimmune diseases; (3) secondary pheochromocytoma induced by other diseases. Two independent investigators screened the literature, extracted data and cross-checked the results, with discrepancies resolved by consensus with a third investigator. Finally, 18 eligible cases were included in the pooled statistical analysis, and the clinical characteristics (age, comorbidities, lesion location) and prognostic outcomes (amputation rate) were summarized and analyzed.

Among 18 similar cases analyzed, (**Supplementary Table 1**), only 4 (22%) presented without hypertension (1), and this case stands out for its 24-year diagnostic delay. This extreme delay highlights the challenge of atypical presentations: when catecholamine excess selectively affects peripheral microcirculation (with absent dorsalis pedis pulses) without inducing central cardiovascular responses, its pathological nature is easily masked as a localized vascular disorder.

A review of global literature over the past 60 years shows common features: patients are typically young (median age 42 years, range 28–76), 38.9% have no underlying diseases, and lower limbs are predominantly affected (upper limb involvement in only 22%). The uniqueness of this case lies in its complex vascular injury pattern—beyond lower limb necrosis, it was accompanied by fluctuating livedo reticularis on the trunk and extremities (**Figure 1b**) a multimodal vasospastic phenomenon described only in the malignant case by Boukhalfa et al. (1). Notably, 38.9% (7/18) of cases in the literature required major amputations due to delayed diagnosis (e.g., below-knee amputation in Lutchman D (2)).In this case, despite active treatment, needed a second toe amputation due to irreversible microcirculatory damage, consistent with the prognosis in Balbir-Gurman et al. (3), underscoring the urgency of early intervention.

The core diagnostic challenge stems from the “decoupling” of pathological mechanisms and clinical manifestations. This case experienced multiple misdiagnoses (including two biopsies that did not suggest tumor association), mirroring the pitfalls in cases reported by Tack et al. (4) (misdiagnosed as diabetic foot) and Kumar et al. (5) (misdiagnosed as Raynaud’s phenomenon). Fundamentally, the false-negative rate of single catecholamine tests can reach 40% (6), and the diagnosis here relied on dynamic biomarker monitoring: peak plasma-free normetanephrine during pain episodes was 17 times higher than in remission. Combined with the 4.8 cm right adrenal tumor found on contrast-enhanced CT, this formed a complete diagnostic evidence chain, providing a new approach to unravel the “occult vasospasm” described by Januszewicz et al. (7).

Long-term follow-up data warn of the far-reaching consequences of delayed treatment: although ulcers healed 1 months postoperatively, the patient still suffers from cold sensitivity and toe loss, consistent with the persistent microcirculatory impairment reported by Bandawar et al. (8). More alarmingly, literature (9) shows an increased risk of catecholamine-induced myocardial infarction in patients with diagnostic delays >10 years (Radtke et al.). Additionally, the malignant recurrence case by Boukhalfa et al. (1)emphasizes the need for lifelong surveillance even with negative genetic screening (5-year recurrence rate of 44% in SDHB mutation carriers).

Based on the clinical characteristics of the present case and the systematic pooled analysis of 18 eligible cases, we put forward three expert opinion-based hypothetical screening proposals for atypical pheochromocytoma in patients with isolated peripheral vascular lesions: ① age <50 years; ② fluctuating acral necrosis; ③ no evidence of atherosclerosis. A combined strategy of dynamic metanephrine monitoring and non-contrast-enhanced MR angiography is recommended to avoid nephrotoxicity, especially for young patients like those in Bessis et al. (10).

This case fundamentally reshapes clinical understanding: acral necrosis may be an early warning sign of catecholamine storm, not a terminal event. Multidisciplinary collaboration (endocrinology for dynamic biomarker testing, vascular surgery for functional assessment, radiology for functional imaging) can significantly reduce the amputation risk from the literature benchmark of 38.9%, providing practical validation for Balbir-Gurman’s (3) “golden intervention window” theory.

## Data Availability

The original contributions presented in the study are included in the article/supplementary material. Further inquiries can be directed to the corresponding authors.

## Supplementary material

SUPPLEMENTARY TABLE 1 Clinical characteristics and prognostic outcomes of 18 cases of atypical pheochromocytoma with isolated peripheral vascular lesions.
