# Supplementary material for: A brief review and case report of pheochromocytoma misdiagnosed as allergic vasculitis with bilateral lower extremity ulcers: a 24-year clinical course
Source: Front Endocrinol (Lausanne). 2026 Apr 10;17:1773861. doi: 10.3389/fendo.2026.1773861 (PMC13106021; doi:10.3389/fendo.2026.1773861)
Supplement: SUPPLEMENTARY TABLE 1 — Clinical characteristics and prognostic outcomes of 18 cases of atypical pheochromocytoma with isolated peripheral vascular lesions. [file Table1.docx]

|  | Author | Age | sex | Tumor Size (cm) | Hypertension | UE Involvement | LE Involvement | Adrena-lectomy | LE Amput-ation | UE Amputa-tion | Exitus |
| --- | --- | --- | --- | --- | --- | --- | --- | --- | --- | --- | --- |
| 1 | This case | 36 | male | 4.8 | No | No | Yes | Yes | No | No | No |
| 2 | Rios et al. | 76 | female | 8 | Yes | Yes | Yes | Yes | Yes | Yes | No |
| 3 | Luchmann et al. | 69 | female | 5 | No | Yes | Yes | Yes | Yes | Yes | No |
| 4 | Balbir-Guman et al. | 63 | female | 4.5 | Yes | No | Right foot | Yes | No | No | No |
| 5 | Bessis et al. | 28 | male | 6 | Yes | No | Left foot | Yes | No | No | No |
| 6 | Tack y Lenders et al. | 41 | female | 5 | Yes | No | Right foot | Yes | No | No | No |
| 7 | Muecrcke y Bliss et al. | 48 | male | 3 | No | No | Left foot | Yes | Yes | No | No |
| 8 | Januszewicz y Wocial et al. | NS/NC | NS/NC | NS/NC | NS/NC | No | Yes | No | No | No | No |
| 9 | Borrega et al. | 37 | female | 5 | Yes | No | Right foot | No | No | No | Yes |
| 10 | Radtke et al. | 59 | female | 5 | Yes | No | Yes | Yes | No | No | No |
| 11 | Radtke et al. | 59 | female | 12 | No | No | Yes | Yes | No | No | No |
| 12 | Scharf et al. | 40 | female | 1kg (autopsy) | Yes | No | Yes | No | No | No | Yes |
| 13 | Scharf et al. | 33 | female | - | Yes | No | Yes | Yes | No | No | Yes |
| 14 | Engelman et al. | 63 | male | - | Yes | Yes | Yes | No | No | No | No |
| 15 | Bandawar et al. | 50 | male | 5.8 | Yes | No | Left foot | Yes | Yes | No | No |
| 16 | Kumar et al. | 28 | female | 4.9 | Yes | Left hand | No | Yes | No | No | No |
| 17 | Mosquera Rey et al. | 57 | male | 4 | Yes | No | Yes | Yes | Yes | No | No |
| 18 | Ahmed Boukhalfa et al. | 42 | male | 11.5 (R)  5.4 (L) | Yes | Yes | Yes | Yes | Yes | No | No |
| LE: lower extremity;UE: upper extremity | | | | | | | | | | | |
